# MANGOU (Miyazaki Advanced New General Surgery of University) Wet Lab Training Relieves Anxiety About Surgical Skills in Surgical Education: A Cross-Sectional Study

**DOI:** 10.7759/cureus.61273

**Published:** 2024-05-28

**Authors:** Masahide Hiyoshi, Kengo Kai, Takashi Wada, Yuki Tsuchimochi, Takahiro Nishida, Takeomi Hamada, Koichi Yano, Naoya Imamura, Fumiaki Kawano, Atsushi Nanashima

**Affiliations:** 1 Division of Hepato-Biliary-Pancreatic Surgery, Department of Surgery, University of Miyazaki, Miyazaki, JPN; 2 Division of Gastrointestinal, Endocrine and Pediatric Surgery, Department of Surgery, University of Miyazaki, Miyazaki, JPN

**Keywords:** willingness to become surgeons, interest in surgery, confidence in surgical skills, questionnaires, surgical education, anxiety, wet lab training

## Abstract

Purpose: To increase the number of medical students or residents who want to become surgeons, we must evaluate our program that recruits new young surgeons.

Methods: We planned surgical training programs for medical students and residents that we named the MANGOU (Miyazaki Advanced New General surgery Of University) training project in the Department of Surgery, Miyazaki University, Japan. From January 2016 through December 2022, we asked trainees who attended this training to complete questionnaires to evaluate their interest in surgery, confidence in surgical skills, and training. Scoring of the questionnaire responses was based on a 5-point Likert scale, and we evaluated this training prospectively.

Results: Among the 109 trainees participating in this training, 61 answered the questionnaires. Two participants found the training boring, but 59 (96.7%) enjoyed it. All of them answered “Yes” to wanting to participate in the next training. Respective pre- and post-training scores were as follows: confidence in surgical skills, 2.2 ± 1.0 and 3.0 ± 1.0 (p < 0.0001); interest in surgery, 4.2 ± 0.8 and 4.4 ± 0.5 (p = 0.0011); and willingness to become surgeons, 3.9 ± 0.7 and 4.1 ± 0.6 (p = 0.0011). All scores rose after MANGOU training.

Conclusion: We planned MANGOU surgical wet lab training for medical students and residents that aimed to educate and recruit new surgeons. After joining the MANGOU training, the trainees’ anxiety about surgery was reduced, their confidence in performing surgical procedures improved, they showed more interest in surgery, and they increased their motivation to become surgeons.

## Introduction

The number of medical students and residents who want to become surgeons is decreasing [1,2]. Problems such as work-life balance [3], risks of litigation [4], and wages [1] were cited when the students considered their future planning and barriers to becoming surgeons. Despite these problems, which are of concern to surgical societies, it is true that not a few young people aspire to and long to become surgeons. However, some of them have no confidence in their surgical technique and skills, think that they would not be a suitable surgeon themselves, and hesitate to become surgeons. We surgeons should help reduce the anxiety of these young people wanting to become surgeons about their surgical skills. Each educational facility offers various programs to attract medical students and residents to surgery. Recently, not only on-the-job training but also educational sessions providing simulator use [5,6], wet lab training [7,8], and cadaver training [9] were highlighted and considered to be useful for surgical training and education.

In this study, we prospectively evaluated the usefulness of our wet lab training as surgical education for medical students and residents from the viewpoint of increasing their interest in surgery and recruitment of surgeons.

## Materials and methods

Miyazaki Advanced New General surgery Of University Project

**Figure 1:**
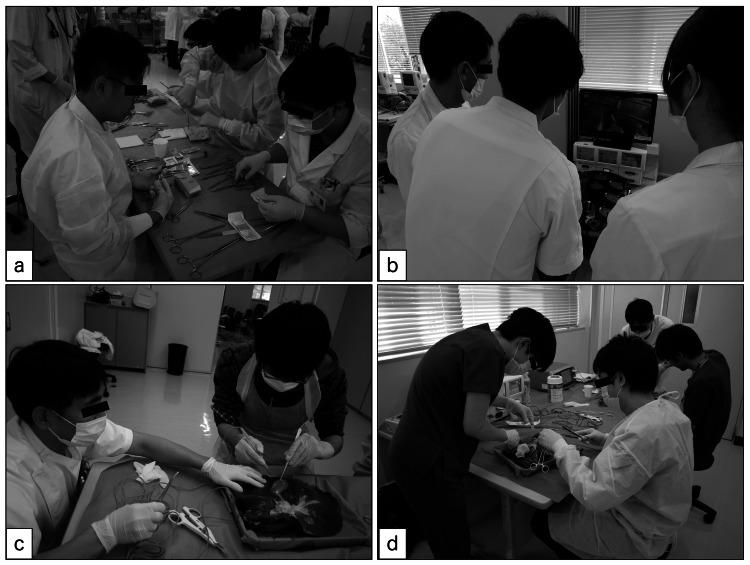
Miyazaki Advanced New General surgery Of University (MANGOU) project We planned surgical training, and especially wet lab training that used animal materials, twice a year as part of the Miyazaki Advanced New General surgery Of University (MANGOU) project. A surgeon is shown teaching surgical procedures such as skin suturing (a), cholecystectomy (b, c), and liver resection (d) to medical students and residents.

We launched surgical training that we call the Miyazaki Advanced New General surgery Of University (MANGOU) project. This project was named after the mango, a specialty fruit of our region. The name was created solely because it is easy for the participants to remember. This project aimed to recruit new surgeons, educate young surgeons, and ensure that surgeons continuously acquire technical skills. We planned the surgical training programs, and especially wet lab training that uses animal materials, twice a year as part of the MANGOU project (Figure 1). This training consists of two components: basic contents such as using electrocautery and skin suturing and advanced contents such as digestive tract anastomosis, cholecystectomy, liver resection, cardiac surgery, lung surgery, and the use of surgical staplers or other surgical devices. The participants attend both basic and advanced training sessions at one time. The contents of the basic course were the same. However, the contents of the advanced course such as liver resection, cardiac surgery, and lung surgery were changed with each training. We performed training in two 90-minute sessions that were announced to medical students and residents using the school’s portal site, job posting poster, and direct verbal communication during the clinical clerkship practicum. The medical students and residents enrolled in the same groups. The groups were made so that the participants with similar clinical years were together. In our institution, five fields of surgery - gastrointestinal, hepato-biliary-pancreatic, cardiovascular, thoracic, and plastic surgery - were combined into one surgery team. Surgeons from these five surgical fields’ participated as instructors in the training.

Target

From January 2016 through December 2022, medical students and residents who participated in this surgical wet lab training and responded to the questionnaire mentioned below were enrolled in this study to evaluate their training. Participation in this training was not obligatory.

Questionnaire

A questionnaire comprising 20 questions was administered before and after the training to the participants using an anonymized form and was evaluated prospectively. We questioned the participants about their degree of interest in surgery, level of confidence in surgical procedures such as skin suturing and intestinal anastomosis, and willingness to become surgeons, how they learned of this training, and their thoughts on participating in training. Responses to the questions were scored on a 5-point Likert scale (Tables 1, 2). The participants answered 11 questions before training (Table 1) and nine questions after training (Table 2). We calculated the point scores of the answers and evaluated the usefulness of this training to increase the participants’ confidence in surgical skills, interest in surgery, and willingness to become surgeons.

**Table 1:** The pre-training questionnaire The participants were asked 11 questions before the training.

Q1. Is this your first time to participate in MANGOU project training?
1 Yes
2 No
Q2. How did you find out about this training?
1 Saw the portal site and bulletin board
2 Saw the poster
3 Via social networking (e.g., LINE)
4 Recommended during internship
5 Word of mouth
Q3. Have you ever sutured skin?
1 No
2 Yes
Q4. (For those answering “Yes” to Q3) Where did you do it?
1 On patients during practical training
2 Using a sponge simulator
3 Using an animal simulator
Q5. Can you suture the skin?
1 I can’t even with guidance
2 I can with guidance
3 I can perfectly with guidance
4 I can on my own
5 I can on my own with confidence
Q6. Have you ever performed anastomosis of the intestinal tract?
1 No
2 Yes
Q7. (For those answering “Yes” to Q6) Where did you do it?
1 In patients during practical training
2 Using a sponge simulator
3 Using an animal simulator
Q8. Can you perform intestinal anastomosis?
1 I can’t even with guidance
2 I can with guidance
3 I can perfectly with guidance
4 I can on my own
5 I can on my own with confidence
Q9. Are you confident in your surgical skills?
1 Not at all
2 Not really
3 I don’t know
4 A little confident
5 Very confident
Q10. Are you interested in surgery?
1 No
2 Not really
3 I don’t know
4 A little interested
5 Very interested
Q11. Do you want to be a surgeon?
1 No
2 Not really
3 I don’t know/may consider other specialties
4 One of my potential choices
5 Yes

**Table 2:** The post-training questionnaire The participants were asked nine questions after the training.

Q12. How was this training?
1 Tiring
2 Neither
3 Fun
Q13. Would you like to participate in the next training?
1 No
2 Not sure
3 Neither
4 Yes
5 Yes, absolutely
Q14. How was the training time (90 minutes per table)?
1 Too long
2 Long
3 Just right
4 Short
5 Too short
Q15. Can you suture the skin?
1 I can’t even with guidance
2 I can with guidance
3 I can perfectly with guidance
4 I can on my own
5 I can on my own with confidence
Q16. Can you perform embedded suturing?
1 I can’t even with guidance
2 I can with guidance
3 I can perfectly with guidance
4 I can on my own
5 I can on my own with confidence
Q17. Can you perform intestinal anastomosis?
1 I can’t even with guidance
2 I can with guidance
3 I can perfectly with guidance
4 I can on my own
5 I can on my own with confidence
Q18. Are you confident in your surgical skills?
1 Not at all
2 No
3 I don’t know
4 A little confident
5 Very confident
Q19. Are you interested in surgery?
1 No
2 Not really
3 I don’t know
4 A little interested
5 Very interested
Q20. Do you want to be a surgeon?
1 No
2 Not really
3 I don’t know/may consider other specialties
4 One of my potential choices
5 Yes

The study protocols were approved by the Human Ethics Review Board of our institution (approval no.: #O-0007; May 10, 2016) and were registered at the University Hospital Medical Information Networks Clinical Trails Registry (UMIN-CTR) as UMIN000052456 (https://center6.umin.ac.jp/cgi-open-bin/ctr/ctr_view.cgi?recptno=R000059871). Agreement by the participants to enter the study was obtained by written consent before they joined the training.

Statistical analysis

All values are expressed as means ± standard error (SE). Statistical analysis by a two-tailed Wilcoxon signed-rank test was performed with JMP software (ver. 16; SAS Institute Inc., Cary, NC). A p value < 0.05 was considered to be statistically significant.

This study has been reported in line with the strengthening of the reporting of cohort, cross-sectional, and case-control studies in surgery (STROCSS) 2021 guideline [10].

## Results

We performed MANGOU surgical wet lab training six times during the study period but could not hold training from 2020 through 2022 due to the COVID-19 pandemic. In total, 109 medical students and residents participated, with 24, 20, 13, 17, 18, and 17 participants attending each of the six training sessions, respectively.

The questionnaire was initiated at the third training session, and 65 participants were enrolled in this study. Of these 65 participants attending the third through sixth training sessions, 61 (93.8%) responded to the questionnaire. Of them, 14 (23.0%) were repeat attendees, and 47 were first-time attendees. These 14 repeat attendees answered the questionnaire for each training. Two participants considered this training to be “boring”, whereas the other 59 (96.7%) enjoyed it. However, all answered “Yes” to wanting to participate in the next training session. Regarding the two 90-minute sessions required for each training, one participant considered it “long”, 35 “just right”, five “short”, and 20 “very short”.

**Figure 2:**
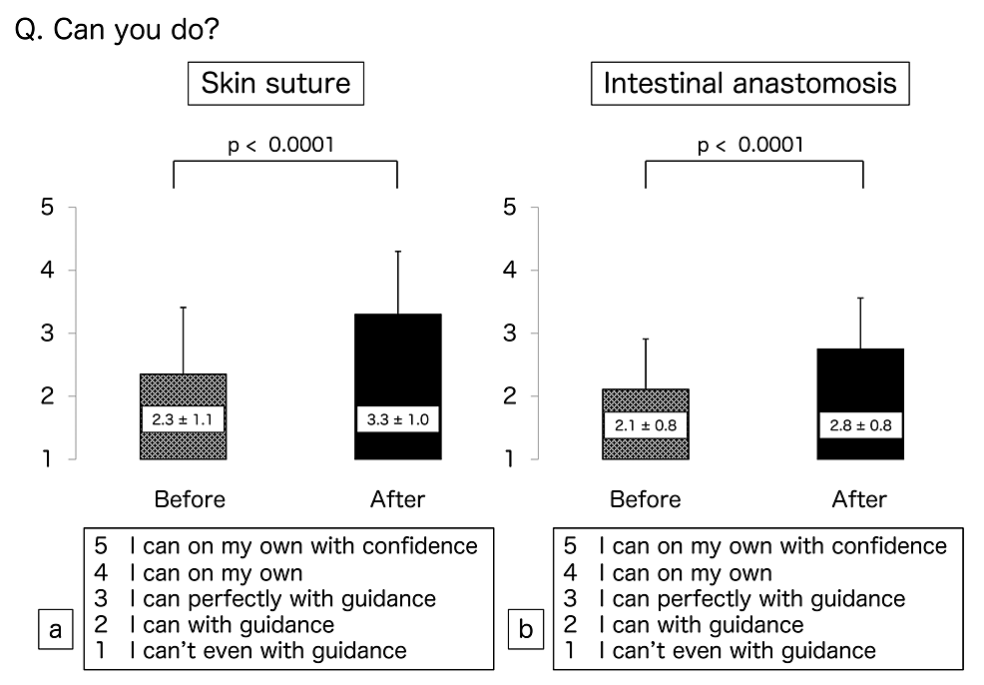
Results of the questionnaires Scoring of the questionnaire responses was based on a 5-point Likert scale. (a) We asked, “Can you suture the skin?” After training, the confidence in skin suture was increased. (b) We asked, “Can you perform intestinal anastomosis?” After training, the confidence in intestinal anastomosis was increased.

**Figure 3:**
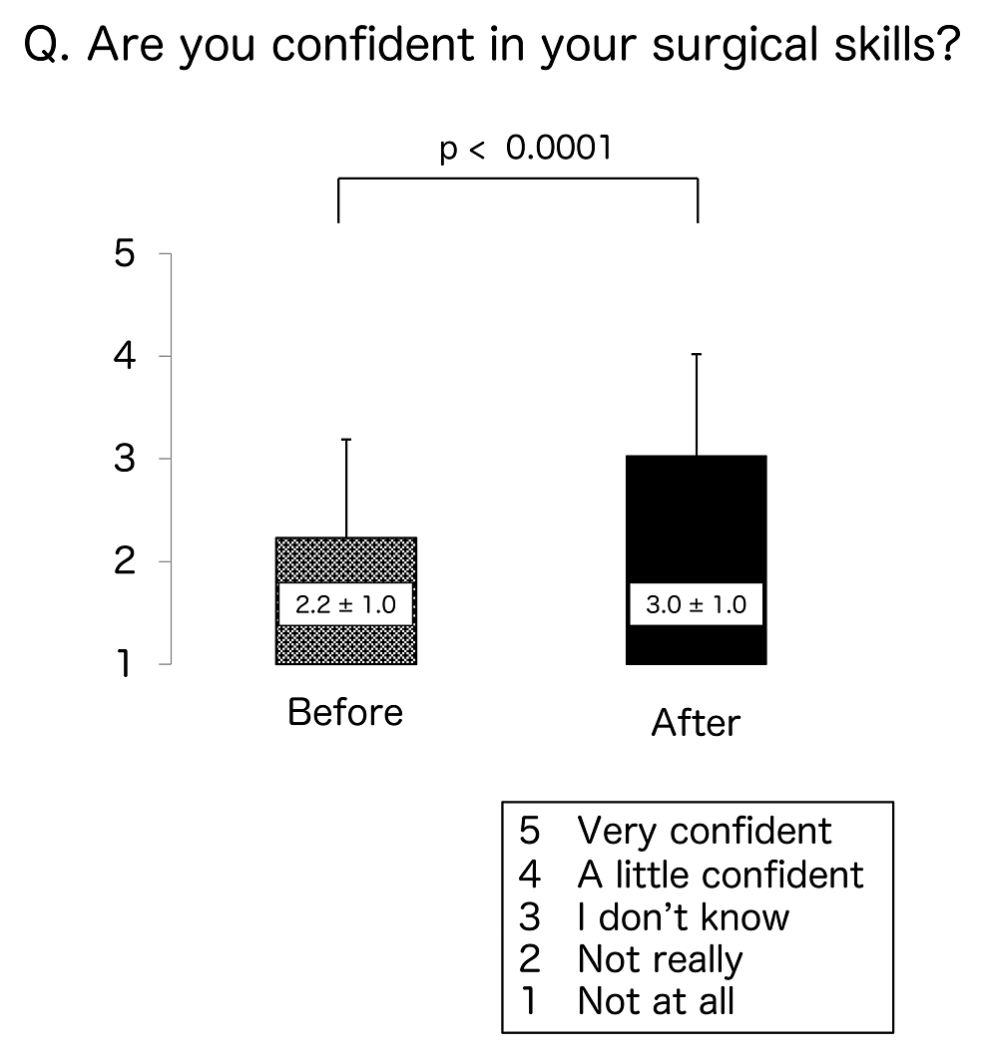
Results of the questionnaires Scoring of the questionnaire responses was based on a 5-point Likert scale. We asked, “Are you confident in your surgical skills?”  After training, the confidence in surgical skills was increased.

**Figure 4:**
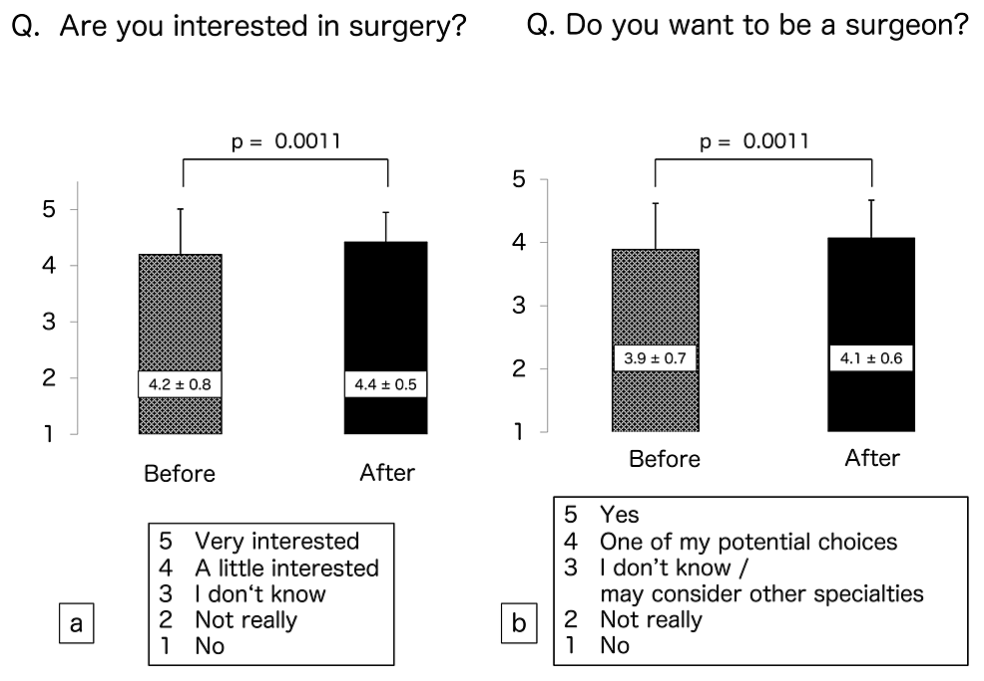
Results of the questionnaires Scoring of the questionnaire responses was based on a 5-point Likert scale. (a) We asked, “Are you interested in surgery?”  After training, the interest in surgery was increased. (b) We asked, “Do you want to be a surgeon?”  After training, the desire to be a surgeon was increased.

The scores of the participants regarding their confidence in performing skin suturing were 2.3 ± 1.1 before training and 3.3 ± 1.0 after training and were significantly different (p < 0.0001) (Figure 2a). Their scores for confidence in performing intestinal anastomosis before and after training were 2.1 ± 0.8 and 2.8 ± 0.8, respectively, and were also significantly different (p < 0.0001) (Figure 2b). Their before and after scores for confidence in overall surgical skills were 2.2 ± 1.0 and 3.0 ± 1.0, respectively (p < 0.0001) (Figure 3). Finally, their respective scores regarding interest in surgery and willingness to become surgeons before and after training were 4.2 ± 0.8 and 4.4 ± 0.5 (p = 0.0011) (Figure 4a) and 3.9 ± 0.7 and 4.1 ± 0.6 (p = 0.0011) (Figure 4b). All scores rose after MANGOU training.

The number of new surgeons recruited to our department from 2016 through 2023 were three, four, three, two, four, three, one, and five, respectively, and they are now working together as surgeons. From 2016 through 2020, which is the period excluding the influence of COVID-19, MANGOU training was conducted normally, and 16 new surgeons were recruited to our department. Of these 16 new surgeons, six (37.5%) had participated in the MANGOU training before recruitment.

## Discussion

The number of medical students or residents who want to become surgeons is decreasing, and this is becoming an alarming problem [1,2], as is the burnout syndrome experienced by experienced surgeons [11,12]. To improve the environment surrounding surgeons, it is very important to recruit new young surgeons. Some trainees feel their surgical technique is not adequate and prevents them from wanting to become surgeons. Thus, various attempts have been reported to reduce their anxiety about surgical techniques so as to recruit and educate new surgeons. Marshall et al. reported that a positive experience during surgical rotations was associated with a higher interest in a career in surgery [13]. Nanashima et al. established a step-by-step surgical education program and reported its usefulness for recruiting young surgeons [14]. Continuous recruiting of new surgeons to prevent burnout of practicing surgeons is important to maintain and develop the various surgical societies.

The MANGOU project was easily accepted, spread, and recognized by medical students and residents in our institution due to its name and the familiarity of mangoes to the trainees. Training or workshops in which surgical techniques can be practiced are useful in improving trainees’ confidence and competence in surgical skills. Clanton et al. reported that a surgical skills workshop for medical students improved their confidence after training [15]. Similarly, our trainees showed confidence in their surgical skills and increased interest in surgery after participating in the MANGOU wet lab training. We found it noteworthy that the trainees participating in this training who were originally interested in surgery were even more interested in surgery and had an increased desire to become surgeons after the training. We assumed that our wet lab training had helped reduce the trainees’ anxiety about surgical procedures and had built their confidence and interest in surgery.

We planned wet lab training that used animal materials for surgical training. Recently, cadaver surgical training has been highlighted and thought to be very useful for trainees [9,16], but ethical issues are involved and it is costly [17]. Therefore, we considered cadaver training not to be suitable for medical students or trainees without basic surgical skills and that it should be provided only to trainees with the minimum required surgical skills and procedural experience. Our goal is to start cadaver surgical training in the future for trainees and practicing surgeons.

Our institution is located in a rural area in which the number of doctors is 246 per 100,000 people, which is the average in Japan. However, fewer people are consistently becoming surgeons, and the population is aging. After participating in our wet lab training, even though the trainees showed greater interest in surgery and a desire to become surgeons, in reality, the number of newly recruited surgeons remained low. The results of the questionnaire did not correlate with the actual recruitment of new surgeons. Against our expectation, this study showed no relation between participants joining this training and whether they became surgeons. Basically, we hire all qualified applicants, and whether they participated in this training did not influence their recruitment. This surgical training relieved the participants from anxiety about surgical skills. However, the number of new surgeons did not increase. To increase the number of new surgeons, we must relieve not only surgical skills but also work-life balance, risk of litigation, and wages.

This study has one important limitation. The questionnaires were completed anonymously, but some of the participants did not respond to the questionnaires out of concern that their responses might affect their academic and training evaluations. Although we explained to them before the training that their responses were anonymous and would have no effect at all on their evaluations, we could not control the opinions of some of the participants.

Finally, one important issue should be considered. In total, 76 surgeons belonging to our department helped with this training as instructors, and we are grateful to the entire surgical staff who cooperated as instructors or made arrangements for this training. That they cooperated without pay and worked on holidays are also problems that we must resolve to improve the environment surrounding surgeons. We think that careful management of working hours, promotion of task shifting, and introduction of incentives are important not only for new surgeons but also for established surgeons and the surgical staff. Proper management of these issues will permit the continued recruiting of new young surgeons and allow them to work with peace of mind and satisfaction, which in turn will benefit their patients.

## Conclusions

We planned MANGOU surgical wet lab training for medical students and residents that aimed to educate and recruit new surgeons. After attending MANGOU training, the trainees’ anxiety about surgery was reduced, their confidence in performing surgical procedures improved, they showed more interest in surgery, and their motivation to become surgeons increased. We will continue this MANGOU surgical wet lab training in order to halt the decrease in the number of surgeons.
